# “Setting people up for success and then failure” – health care and service providers’ experiences of using prize-based contingency management

**DOI:** 10.1186/s13011-020-00316-z

**Published:** 2020-09-14

**Authors:** Marilou Gagnon, Adrian Guta, Alayna Payne

**Affiliations:** 1 Canadian Institute for Substance Use Research, University of Victoria, 2300 McKenzie Ave, Victoria, BC V8N 5M8 Canada; 2 School of Social Work, University of Windsor, 167 Ferry Street, Windsor, ON N9A 0C5, V6T2B5 Canada

**Keywords:** Contingency management, Drugs, HIV, Incentives, Providers, Qualitative, Substance use

## Abstract

**Background:**

Over the last 50 years, there has been a growing interest in and use of contingency management (CM) for people who use substances. Yet, despite showing some level of efficacy (albeit only short-term) and being praised by researchers as beneficial and cost-saving, it continues to be underutilized by health care and service providers. Why that is remains unclear.

**Methods:**

Recognizing a gap, we conducted a targeted analysis of a larger set of qualitative interviews conducted on the experience of health care and service providers with incentives (including prize-based CM) (n = 25).

**Results:**

Four themes were identified during the analysis: 1) The specificities of prize-based CM, 2) The role of providers in administering prize-based CM, 3) The positive and negative impact on the relationship, and 4) The ethical concerns arising from prize-based CM. Overall, our findings are consistent with existing literature and suggest that providers are wary of using prize-based CM because they tend to value effort over success, support over reward, honesty over deceit, and certainty over probability and variability.

**Conclusion:**

Our analysis offers additional insights into the experiences of providers who use prize-based CM and possibly some indications as to why they may not wish to work with this type of incentive. The question raised here is not whether there is enough evidence on the effectiveness of prize-based CM, but rather if this type of incentive is appropriate and ethical when caring for people who use substances.

## Background

Over the last 50 years, there has been a growing interest in and use of contingency management (CM) for people who use substances [1–5]. CM is rooted in operant conditioning (also known as instrumental conditioning) and based on the general principle that behaviors, such as substance use, are controlled by their consequences [6]. To modify such behaviors, one has to modify the consequences using positive or negative reinforcements [3]. When used with people who use substances, CM is typically designed to provide positive reinforcements in exchange for desired processes (e.g., attending a clinic appointment), behaviors (e.g., maintaining abstinence), and outcomes (e.g., negative urine drug screening) [1, 3, 4, 7].

Two types of positive reinforcements are most commonly used, voucher-based CM and prize-based CM [7]. Voucher-based CM provides set amounts in the form of vouchers that can be exchanged for goods or services [7]. However, this type of CM intervention is too expensive [6]. Prize-based CM, also called fishbowl CM, tries to lower the costs by incorporating probability and variability [7]. A standard fishbowl contains 500 slips with about half of the slips being non-winning and featuring messages such as “good job” [7]. The remaining slips are divided as follows: the majority of the slips are valued at $1, a small number of slips are valued at $20, and there is invariably only one large value slip of $100 [7]. Similar to voucher-based CM, slips can be exchanged for goods and services or exchange for a gift card of the same value amount.

Prize-based CM has been studied among people using stimulants, opioids, cannabis, nicotine, benzodiazepines, alcohol, as well as people who use multiple substances [8]. Yet, despite showing some level of efficacy (albeit only short-term) and being praised by researchers as beneficial and cost-saving, it continues to be “the least implemented” of all empirically-based interventions in substance use [8]. This is, in part, due to overall cost and logistics of implementing prize-based CM, but it is also reflective of a lack in awareness and knowledge on the part of health care providers as well as their overall weariness in adopting this type of incentive [7, 8]. Surprisingly, very few studies have explored the perspective of health care and service providers. Recognizing a gap, and building on our shared experiences as services providers and researchers in the field of substance use, we conducted a targeted analysis of a larger set of qualitative interviews conducted on the experience of health care and service providers with incentives (including prize-based CM).

## Methods

To explore the experience of health care providers with incentives, including but not limited to prize-based CM, we conducted a qualitative case study [9, 10]. We focused specifically on the care of people at risk and living with HIV because there has been a steady growth of incentives across the fields of HIV prevention, testing, treatment, and care. We also selected the province of British Colombia because it has a long tradition of using incentives in the care of people at risk or living with HIV who are hard-to-reach, including people who use substances.

### Data collection

Health care providers (i.e., physicians, nurses, social workers) and service providers (i.e., community-based workers, peer workers) who work in not-for-profit community-based organizations, health centres, and HIV clinics or programs were recruited using email invitations and recruitment e-cards. We also sent email invitations to existing networks of health care and service providers. Participants were eligible to take part in this study if they: identified as a health care or service provider; had worked with people at risk or living with HIV in the past five years; and had a least one experience working with incentives. The lead researcher (M.G.) conducted semi-structured in-depth interviews with 25 providers (Table 1), lasting on average 45 to 90 min. Informed consent was obtained before each interview. Interviews focused on the actual hands-on experience of working with incentives with particular emphasis on context, role, use, benefits, limitations, ethical tensions, and broader implications (see Table 2). All interviews were audio-recorded, transcribed, and analyzed.

**Table 1:** Sample characteristics

What year were you born?	
1950s-1960s	4
1970s-1980s	15
1990s	6
How would you describe yourself?	
Man	5
Woman	19
Non-binary	1
What is your highest level of education?	
High School	2
College	6
Undergraduate	16
Graduate	1
What is your position?	
Registered Nurse	15
Social Worker	2
Community Worker	3
Peer Worker	5
How long have you worked in this position?	
< 5 years	6
5–10 years	14
> 10 years	5
How long have you worked with people at risk or living with HIV?	
< 5 years	6
5–10 years	15
> 10 years	4

This table provides a description of our participants including age, gender, highest level of education, professional position, years of experience in the position, and years of experience working with people at risk and / or living with HIV

**Table 2:** Interview guide

Background context	1. Please describe your current role
	a. What is your training and background?
	b. What are your main responsibilities?
	c. How long have you been working in this role?
	2. What happens in a typical day for you?
	a. Who do you see?
	b. Who do you interact with?
Using incentives	1. Please tell me about your experience with incentives
	a. What was the context
	b. How were you involved in the process or implementation?
	c. What were the overall objectives?
	2. Please tell me about how incentives were used in the program
	a. What was your role?
	b. How were you supported in this role?
	3. How did your colleagues respond to the use of incentives?
	a. Can you share examples of positive and negative responses?
	4. How did the patients/clients respond to the use of incentives?
	a. Can you share examples of positive and negative responses?
	b. Did any issue arise? If so which one and how were they addressed?
	5. How did incentives impact your practice/service?
Incentives broadly	1. Based on your experience, what were the benefits of using incentives?
	2. Based on your experience, what were the challenges of using incentives?
	3. What role do you think incentives should have in health care?
	4. How can we ensure incentives are provided equitably?
	5. How can we best support health care and service providers?
	a) What are the current gaps? (e.g., training, support, best practices)
	b) How and who should we address these gaps?

This table summarizes the key questions asked during the interview

### Data analysis

To analyze the participant interviews, we used Applied Thematic Analysis (ATA) [11]. In summary, ATA involves four general steps: 1) read and code transcriptions, 2) identify possible themes, 3) compare and contrast themes, identifying structure among them, and 4) produce a thematic scheme to describe the research phenomenon.

To complete the first round of analysis and develop a matrix, the lead researcher (M.G.) analyzed six interviews that were selected based on data richness, diversity of experiences, and completeness (i.e., based on the interview guide). The matrix included six high-level themes, which emerged from the six interviews and reflected the interview guide, namely, 1) context, 2) goals, 3) incentives, 4) successes, 5) limitations, and 6) strategies. These themes were discussed with and approved by the co-lead researcher (A.G.) over the course of an analytic debrief meeting. To ensure that the matrix developed over the course of the first round was indeed complete, the same six interviews were analyzed a second time by a research assistant (A.P.). Using the same matrix, the remaining interviews were analyzed in NVivo. This matrix allowed us to work systematically through large amounts of data while keeping the focus of the analysis on specific content areas and identifying emerging themes. As the process evolved, we were able to refine the themes and identify sub-themes in order to develop a more nuanced, structured, understanding of the findings. Findings of this analysis have been presented elsewhere [12].

To explore the experience of providers with prize-based CM more specifically, we conducted a targeted analysis using applied thematic analysis again [11]. First, a research assistant (A.P.) screened the interviews and extracted content related to this particular type of incentive. 5 of the 25 providers had worked with prize-based CM, representing 20% of our sample. Then, all three members of the research team worked together to identify recurrent themes across the 5 interviews. Four themes were identified 1) The specificities of prize-based CM, 2) The role of providers in administering prize-based CM, 3) The positive and negative impact on the relationship, and 4) The ethical concerns arising from prize-based CM.

## Results

Our participants had multiple experiences of using incentives in various roles and in various settings. Those who had hands-on experiences with prize-based CM all used the same standard intervention whereby clients were asked to draw from a standard fish bowl as described above. This particular type of incentive was used specifically with clients who used substances and clients who struggled with treatment adherence to reward processes (e.g., attending a group session or clinic appointments), behaviors (e.g., maintaining abstinence or treatment adherence), and outcomes (e.g., negative urine drug screening or undetectable viral load).

### The specificities of prize-based CM

Providers found that prize-based CM differed from other types of incentives on at least two levels. First, they pointed out that the incentive is contingent upon succeeding not trying. As such, the draw – and more importantly the probabilistic chance of winning the large value $100 slip – acts as a reward and makes the achievement of the target process, behavior, and outcome more appealing despite the actual monetary value of the incentive being quite low. Second, they noted that it worked in a similar way as gambling by leveraging the desire to draw the biggest slip. Participants referred to this as “slot machine theory”. As one participant puts it:

Contingency management was like an incentive but also based on “slot machine theory” where you keep coming back because you don’t know if you’re going to get that $100 or nothing if you manage to meet your goals. (Participant 5).

### The role of providers in administering prize-based CM

Providers were tasked with administering prize-based CM. Concretely, the described their role as having to ensure that the right clients were enrolled, assessing if clients were indeed meeting target processes, behaviors, and outcomes, and granting or refusing permission to draw from the fishbowl. Taking on this role meant they had to communicate and manage expectations around the draw as well as establish and maintain strong boundaries to make sure rules were followed (i.e., draw upon success nothing less). It also meant they had to experience the highs and more often than not the lows of the fishbowl along with their clients.

You see the excitement that someone is able to do the fishbowl and how exciting that is and it was exciting for me as well. So I would experience that disappointment as well. Like I would feel terrible. It’s like, you have been doing this for weeks and weeks and weeks and then you get like $5? (Participant 4).

### The positive and negative impact on the relationship

According to providers, prize-based CM had some positive impact on their relationship with clients by improving engagement and the feeling of being valued. However, they also saw some negative impact because it is designed to be deceptive. In other words, it is designed to make clients think they will win large sums of money it they succeed when in fact the probability of that happening is extremely low. Providers worried about the impact of repetitive disappointment from either winning low-value slips (or non-winning slips) or being denied the opportunity to draw. As such, they worked to “support clients through their own disappointment” (Participant 16) and to minimize the potential harms of clients feeling “betrayed”.

### The ethical concerns arising from prize-based CM

The main ethical concerns raised by providers pertained to fairness, autonomy, power imbalance, and effectiveness. Fairness came up as an issue since prize-based CM is a targeted, not universal, incentive. Providers mentioned that clients who were not eligible often raised this sense of unfairness with them, with some suggesting that they would go off their medications to become eligible. Autonomy was also an issue because of the powerful appeal of prize-based CM. This made the process of obtaining free and informed consent challenging for providers. Furthermore, there was a greater sense of power imbalance when working with prize-based CM because of the role providers played and the power they exercised in administering this type of incentive. Finally, the short-term effectiveness of prize-based CM raised some additional ethical concerns. As one provider puts it:

Just from the data we took from it and from the experience, I don’t think [prize-based CM] is worth it. I think we can utilize other engagement tools (…) I think in some cases there is a place for incentives, but I also think that it can raise issues when they are taken away and clients stop adhering to their treatment or whatever they are being incentivized to do (…) The thought of setting people up for success and then failure when you withdraw the incentive is not a nice thing to do. (Participant 5).

## Discussion

Our findings suggest that prize-based CM can have a powerful effect on clients who experience the greatest challenges in meeting target processes (e.g., attending a group session or clinic appointments), behaviors (e.g., maintaining abstinence or treatment adherence), and outcomes (e.g., negative urine drug screening or undetectable viral load). However, as pointed out by providers, its effect decreases over time as clients realize that the probability of winning large sums of money is in fact, lower than expected. Its effect also stops when the draws stops. To put it simply, prize-based CM works as long as the *appeal of winning* motivates clients. If the appeal fades away or disappears, clients go back to their baseline struggles. This raises important limitations and ethical concerns that may affect providers’ willingness to use prize-based CM.

Acceptability studies suggest that providers are more inclined to support universal as opposed to targeted incentives [13, 14]. They are also more likely to support incentives offered with the greatest level of certainty (i.e., clients know with certainty that they will receive a fixed incentive upon meeting a target) [13, 14]. Finally, they are more willing to support incentives that optimize health and yield maximum effect over time versus incentives that encourage “gaming” such as prize-based CM [13, 14]. Our findings are consistent with this body of literature and suggest that providers value effort over success, support over reward, honesty over deceit, and certainty over probability and variability.

Our findings are also consistent with studies conducted on the experiences of providers who implement prize-based CM. These studies raise a number of issues that echo what we found during our analysis including but not limited to the 1) overemphasis on abstinence rather than harm reduction; 2) high operational costs and low reward for clients; 3) concerns about fairness, coercion, motivation, and effectiveness; 4) impact on the provider–client relationship; and 5) added roles and responsibilities [15–19]. Unlike previous studies, however, we found that providers called into question the core principle of prize-based CM, which is to capitalize on the *appeal of winning* (much like gambling) in order produce the greatest results at the lowest cost. Essentially, this type of incentive asks “how low can we go, and with whom?” [20]. The question raised by our findings is two-fold.^1^ First, what are the ethical implications of using an incentive that mimics gambling when providing care to people who use substances when we know that both behaviors share a common neurobiological pathway? Specifically, what are the implications of increasing dopamine activity simply to maximize response and lower cost?

Our study has limitations stemming from the fact that it was derived from a larger set of qualitative interviews conducted on the experience of health care and service providers with incentives – inclusive of but not specific to prize-based CM. As such, we did not gather specific information about the PB-CM intervention design (e.g., length of the intervention) and instead focused more broadly on the experience of providers tasked with using prize-based-CM. Furthermore, our sample is limited to 25 providers recruited from a specific geographic area, which is appropriate for a qualitative case study but may limit generalizability. As previously mentioned, prize-based CM continues to be “the least implemented” of all empirically-based interventions in substance use [8] and there is not a large pool of health and service providers who have used prize-based CM in practice. Our study addresses this evidence gap through rich data and diverse perspectives with representation from social workers, nurses, community workers, and peer workers. Furthermore, it provides critical insights into a practice that is not widely implemented but is gaining traction in Canada and internationally. We believe that our findings, generated from a small sample and in a geographically limited area, may serve as the basis of future research with larger samples (e.g., anonymous national and international surveys which might enable more practitioners to participate and share their experiences, concerns, etc.) and help inform practice-based discussions as the interest in and uptake of prize-based CM continues to grow in the upcoming years.

## Conclusion

Our analysis offers additional insights into the experiences of providers who use prize-based CM and possibly some indications as to why they may not wish to work with this type of incentive. The question raised here is not whether there is enough evidence on the effectiveness of prize-based CM, but rather if this type of incentive is appropriate and ethical when caring for people who use substances.

## Data Availability

In order to protect the confidentiality and anonymity of participants, the data (transcripts) will not be shared.

## Abbreviations

CM: Contingency Management
HIV: : Human Immunodeficiency Virus

## Footnotes

1 Additional background to help unpack this question: The dopamine system is associated with behaviors such as substance use and gambling [21]. Studies have found that people with lower dopamine activity or people with reduced dopamine release capacity are most likely to be *non-responders* to prize-based CM [2]. Furthermore, research has shown that dopamine release capacity outperformed other predictors of prize-based CM response such as demographic or substance use severity [2]. In their systematic review, Forster et al. [2] conclude that more research is needed to understand the role played by dopamine in predicting individual responses and understanding individual differences in prize-based CM response. Furthermore, although studies have demonstrated that prize-based CM does not inherently increase gambling tendencies, gambling tendencies or being in recovery from pathological gambling are standard exclusionary criteria for prize-based CM studies.
